# Patient empowerment through a user-centered design of an electronic personal health record: a qualitative study of user requirements in chronic kidney disease

**DOI:** 10.1186/s12911-021-01689-2

**Published:** 2021-11-24

**Authors:** Esmaeel Toni, Habibollah Pirnejad, Khadijeh Makhdoomi, Azam Mivefroshan, Zahra Niazkhani

**Affiliations:** 1 Student Research Committee, Urmia University of Medical Sciences, Urmia, Iran; 2 Department of Health Information Technology, Urmia University of Medical Sciences, Urmia, Iran; 3 Patient Safety Research Center, Clinical Research Institute, Urmia University of Medical Sciences, Urmia, Iran; 4 Erasmus School of Health Policy & Management (ESHPM), Erasmus University Rotterdam, Rotterdam, The Netherlands; 5 Department of Adult Nephrology, Urmia University of Medical Sciences, Urmia, Iran; 6 Nephrology and Kidney Transplant Research Center, Clinical Research Institute, Urmia University of Medical Sciences, Urmia, Iran

**Keywords:** ePHR, Electronic personal health record, User requirements, Self-management, Developing country, Chronic kidney disease, CKD, Nephrology, Chronic care, Chronic disease

## Abstract

**Background:**

To improve chronic disease outcomes, self-management is an effective strategy. An electronic personal health record (ePHR) is a promising tool with the potential to support chronic patient’s education, counseling, and self-management. Fitting ePHRs within the daily practices of chronic care providers and chronic patients requires user-centered design approaches. We aimed to understand users’ needs and requirements in chronic kidney disease (CKD) care to consider in the design of an ePHR to facilitate its implementation, adoption, and use.

**Methods:**

A qualitative study was conducted in a major Iranian nephrology center including inpatient and outpatient settings in 2019. We conducted 28 semi-structured interviews with CKD patients, nurses, and adult nephrologists. To confirm or modify the requirements extracted from the interviews, a focus group was also held. Data were analyzed to extract especially those requirements that can facilitate implementation, adoption, and sustained use based on the PHR adoption model and the unified theory of acceptance and use of technology.

**Results:**

Participants requested an ePHR that provides access to up to date patient information, facilitates patient-provider communication, and increases awareness about patient individualized conditions. Participants expected a system that is able to cater to low patient e-health literacy and high provider workload. They requested the ePHR to include purposeful documentation of medical history, diagnostic and therapeutic procedures, tailored educational content, and scheduled care reminders. Messaging function, tailored educational content to individual patients’ conditions, and controlled access to information were highly valued in order to facilitate its implementation, adoption, and use.

**Conclusions:**

We focused on the ePHR’s content and functionalities in the face of facilitators and/or barriers envisioned for its adoption in nephrology care. Designers and implementers should value CKD patients’ needs and requirements for self-management such as providing personalized education and counseling (on the basis of their condition and risk factors), health literacy, and disease progression levels. The socio-technical aspects of care also need further attention to facilitate ePHR’s adoption.

**Supplementary Information:**

The online version contains supplementary material available at 10.1186/s12911-021-01689-2.

## Background

Self-management is an effective strategy to improve chronic disease patient’s clinical outcomes and quality of life [1]. As information technologies facilitate access to health information, their use can increase patient’s health knowledge and enable patient-centered approaches to the delivery of care [2]. Electronic personal health records (ePHRs) are seen as one of the most promising tools to support patient’s self-management [3, 4]. They improve patient-provider communication and empower patients to self-manage their diseases, effectively [5]. However, implementing an ePHR is not an easy task even in the developed countries due to various barriers [6, 7]. For example, the experience of implementing an ePHR in the United Kingdom showed that poor attention to user expectations and weak system usability led to the system’s low adoption [8]. In order to be widely used, ePHRs should meet different users’ expectations regarding patients’ conditions and fit in the existing health and technology infrastructures [9]. Therefore, for their successful deployment, one needs to pay attention to the user-centric design approaches of ePHRs while having an eye on the available infrastructure [10].

According to the 2017 global burden of disease, the prevalence of chronic kidney disease (CKD) is continually rising [11]. CKD is a worldwide health concern that is defined as the gradual loss of kidney function and divided into five stages based on the estimated glomerular filtration rate (eGFR), with each stage requiring especial set of CKD care [12]. As the disease progresses, the stage of the disease increases, so that the stages 4 and 5 are called end-stage renal disease (ESRD) requiring expensive renal replacement therapies of dialysis or kidney transplantations [13]. The rising healthcare expenditure on complex CKD care requires innovative approaches to contain cost while maintaining the quality of care [14]. Self-management in CKD care has been beneficial; it includes interventions related to lifestyle modifications (e.g., targeting nutrition, weight management, and physical exercise) and medical-behavior modification (e.g., targeting medicine adherence, disease cognition, and complication control) [15, 16]. To support self-management, many researchers have aimed to develop ePHRs for CKD patients in order to enable them to play an active role in their own care by facilitating more productive communication and interaction with care providers [17–19]. To this end, ePHRs need to satisfy individualized needs and requirements in different CKD stages and fit in with the daily practice of care providers and the healthcare context and infrastructure [20–22]. Yet, most literature is from the developed countries and little experience has been shared from the developing countries. Therefore, as a first step to develop an ePHR for CKD in a developing country, we aimed to have a better understanding of users’ needs and requirements in their own care context. We were interested in getting this insight in the face of an ePHR's likely adoption facilitators and/or barriers and consider them in its design and implementation.

## Methods

### Study design

This was a qualitative study conducted in a major nephrology care center in an academic hospital of Urmia University of Medical Sciences (UUMS), in Iran. The study setting included nephrology inpatient wards, outpatient clinics, and dialysis centers (i.e. hemodialysis and peritoneal dialysis units). All units use a hospital information system (HIS). Recently, a CKD disease registry has been implemented in nephrology care. The study was approved by the Ethical and Research Committee of the UUMS.

### Recruitment and participants

In this setting, we conducted a qualitative study and collected data through in-depth, face-to-face, semi-structured, and one-on-one interviews as well as a focus group performed between February to August 2019. Participants were selected through “purposive” sampling [23] to identify the requirements for an ePHR for CKD patients. We selected the patient participants according to the following inclusion criteria: (a) being a confirmed CKD patient (with different underlying diseases and comorbidities), (b) being in different phases of the disease progression (i.e., CKD stages 1–5, before or after starting the dialysis), and (c) being at least 18 years old or above. We excluded patients who were (a) under 18 years old, (b) very ill, or (c) uninterested to interview with us. In few cases, patients' care givers also participated in the interview session in patients' attendance. We invited all nurses who worked in the dialysis units and nephrology inpatient and outpatient settings for participation in our study. Also, all adult nephrologists who involved in the care of CKD patients in our study setting were invited for participation. Pediatrics nephrologists were excluded because our focus was on adult nephrology care.

In the first phase of this study, we interviewed CKD patients, nurses, and adult nephrologists. All nephrologists involved in nephrology care at the time of this study were invited to participate; all participated, except one. We recruited patients with consideration to their gender, age, social status, educational level, stages of the disease, and hospital settings. Some of the patients and most of nurses were proposed by our participating nephrologists as informant patients and nurses. We stopped recruiting interviewees when the saturation of data in each group of nurses and patient participants was reached.

### Data collection

The interview questions were designed according to the knowledge and roles of the three participant groups. Additional file 1: Appendix 1 provides the details of questions in our study. Formal consent was sought from the participants. They either completed a written consent form or gave verbal consent to participate in the interviews. The interviews (lasting on average 25 min) were audio-recorded after assuring participants that their confidentiality would be respected by the researchers. The interviews were transcribed verbatim. To ensure the validity of data, "member checking" was performed with the clinicians to confirm or correct their statements.

Afterward, we held a focus group with clinicians to discuss, confirm, or modify the information extracted from the analysis of the interviews. Like interviewees, the participants were purposefully selected. Some of them had previously participated in our interviews, as well. The focus group lasted for 1 h 45 min. During the focus group, a moderator (the corresponding author) and a note keeper (the first author) managed and facilitated the discussions among participants based on our questions (Additional file 1: Appendix 1). This session helped to reach a consensus on the scope of the PHR and its functionalities (based on the requirements extracted from interviews) that would work in our context. Complemented by the written notes taken by the note keeper, the discussions were recorded and transcribed verbatim.

### Data analysis and assessment

We used the “Personal Health Record Adoption Model” (PHRAM) to analyze the ePHR requirements and envisioned facilitators/barriers mentioned by CKD Patients for ePHR adoption [24]. This model integrates five factors that interact to affect the intent to adopt, and ultimately use, PHRs. These factors are personal (including demographics, e-health literacy, self-efficacy, knowledge, and skills), environmental (including facilitating conditions and incentive motivation), technology (including cost, perceived usefulness, perception of external control, relative advantage, and complexity), and chronic disease factors (including self-management, preferences for self-regulation, perceived complexity of condition and treatment, access to care, and multiplicity of settings, healthcare providers, and chronic illnesses) that affect the behavioral outcome (i.e., in our study intention to adopt a given ePHR). For the analysis of the requirements requested by our care providers and envisioned facilitators/barriers for an ePHR’s adoption in CKD care, the “Unified Theory of Acceptance and Use of Technology” (UTAUT) was applied [25]. The UTAUT comprises of four main factors of performance expectancy, social influence, effort expectancy, and facilitating conditions, which have prominent roles as direct determinants of user acceptance of a technology and usage behavior.

In order to address our study aims, the interview questions (Additional file 1: Appendix 1) and the following discussion on the interviewers' responses was guided to cover the factors in the two aforementioned frameworks as far as possible. The transcribed data were imported into the AtlasTi (ATLAS.ti 8, Berlin, Germany). The first author systematically coded the data to identify and categorize key themes. The corresponding author closely supervised this process and double-checked the extraction of themes based on the two aforementioned frameworks as well as the whole analysis. The inter-coder reliability was 0.84 between these two coders. Any disagreement was solved by discussion during multiple meetings. An audit log of decisions was kept along with the codes.

## Results

### Study participants

We undertook 28 interviews with 15 CKD patients, 10 nephrology nurses, and three adult nephrologists. Patients’ age ranged 21–79 years; six patient participants were at the early CKD stages, and almost equally recruited from inpatients, outpatients, hemodialysis, and peritoneal dialysis settings. Five nurses from the nephrology and the dialysis units and one senior nephrologist participated in our focus group meeting. Table 1 provides the details of our participants. The results are provided here based on the factors in the PHRAM and UTAUT models. Some exemplary quotes based on these factors, indicated with (Q#) within the text, are available in Table 2.^1^

**Table 1:** Demographics of the study participants (n = 28)

	Patients (n = 15)	Nurses (n = 10)	Nephrologists (n = 3)
Gender	9 males	1 male	0 male
	6 females	9 females	3 females
Mean age (years)	50.73 (range 21–79)	31.6 (range 22–47)	46 (range 40–55)
*Age group N (%)*			
20–29	3 (20)	4 (40)	0 (0)
30–39	1 (6.6)	5 (50)	0 (0)
40–49	3 (20)	1 (10)	2 (66.6)
50–59	3 (20)	0 (0)	1 (33.3)
60–69	2 (13.3)	0 (0)	0 (0)
70–79	3 (20)	0 (0)	0 (0)
Mean years since diagnosis^*^	6.26 (range 0–25)	N/A	N/A
Years since diagnosis N (%)*			
< 5	9 (60)	N/A	N/A
5–10	3 (20)		
11–15	2 (13.3)		
16–20	0 (0)		
21–25	1 (6.6)		
Mean years of professional experience	N/A	9.1 (range 0–26)	11.66 (range 6–22)
*Years of professional experience N (%)*			
< 5	N/A	4 (40)	0 (0)
5–10		2 (20)	2 (66.6)
11–15		2 (20)	0 (0)
16–20		1 (10)	0 (0)
21–25		0 (0)	1 (33.3)
26–30		1 (10)	0 (0)
Hospital sections of participant sampling	4 patients in outpatient CKD clinic	4 nurses in nephrology inpatient unit	3 adult nephrologists covering nephrology units across the hospital
	3 patients in nephrology inpatient unit	5 nurses in hemodialysis unit	
	5 patients in hemodialysis unit	1 nurse in peritoneal dialysis unit	
	3 patients in peritoneal dialysis unit		
Education	3 patients with middle school and lower education	10 nurses with a bachelor degree	3 adult nephrology specialists
	8 patients with high school education		
	3 patients with a bachelor degree		
	1 patient with a master's degree		
Stage of the disease*	6 patients with CKD stages 1–3	N/A	N/A
	9 patients with CKD stages 4–5		

*N/A* not applicable

*Only applicable for the patient participants

**Table 2:** The PHRAM and UTAUT constructs with the corresponding quotes form our study participants

Model	Themes/sub-themes	Participant quotes
**PHRAM**	**Personal factors**	
	Self-efficacy	Q1: “Well, [with access to an ePHR] I can find out more about my disease… [Also] I will be able to control my own condition more easily and take care of my own health… I can make better decisions about taking care of myself outside the hospital and be aware of the information in my [medical] record.” *(A stage 2 CKD patient)*
	Communication preferences	Q2: “Sometimes there is discharge, swelling, etc. around my abdomen wounds, and I do not know if those conditions are not important or I must come to Urmia from another city for treatment. For example, if it is possible for me to take a photo from the wound [to share it with my doctor through an ePHR] and my doctor says whether or not I need to come to Urmia, it will be great.” *(A stage 5 peritoneal dialysis patient)*
	Knowledge	Q3: “[Using an ePHR,] I can quickly access the right information. For example, I can be informed about which foods have the highest amount of potassium and how much I can consume them… or what are the side effects for a higher consumption of phosphorus in my diet?” *(A stage 5 patient under hemodialysis)*
	Outcome expectations	Q4: “As a matter of fact, it [an ePHR] is very good for some patients who are in the early stages and have much stress, because they do not know much about their disease.” *(A nephrologists)* Q5:“ If the patient wants to go to another city to continue the treatment process and have access to the [ePHR] system, it will give them [care providers] more information [about the patient] and the patient will pay less for the diagnostic tests.” *(A nurse)*
	Skills	Q6: “… very good, then I can control my health [condition]. For example, how much is my creatinine and what can I do to decrease it? It gives me all of my personal information, I can improve my care performance… my life performance will be improved [, too]”. *(A stage 5 CKD patient under hemodialysis)*
	Demographics	Q7: “… Many patients are old or illiterate and this will prevent them from accessing the [ePHR] system.” *(A stage 1 CKD patient)*
	Attitude	Q8: “Maybe some patients do not spend time on such things [ePHRs] or do not pay attention to them.” *(A stage 5 CKD patient under hemodialysis)*
	e-health literacy	Q9: “Some patients cannot use or access the Internet and do not have enough medical information. It should be in a simple language that everyone can use.” *(A stage 2 CKD patient)* Q10: “… not all of them [patients] are educated. Also, some of our patients might not be able to use [an ePHR] … patients can't record everything [in the system by themselves], for example, patients can't record their CT-scan [result that] “hydronephrosis” was observed in my left kidney”, our patients can't record these types of information …” *(A nurse)*
	**Environmental factors**	
	Facilitating conditions	Q11: “…I'm an educated person, but, really, I don't know how to work with the internet. I think it is difficult to learn. If my daughter works with an ePHR instead of me, that will be fine because she knows how to work with it…”. *(A stage 5 peritoneal dialysis patient)*
	Incentive motivation	Q12: “For example, I drink coffee or usually smoke every morning. This [ePHR] should be able to remind me or warn me … because I usually forget these things … I think if this [such feature] is available I will take more care of myself …” *(A stage 2 CKD patient)*
	Social influence	Q13: “This [the use of ePHR] should be authorized by physicians and supported by them because the patient trusts her doctor……” (A care giver of a stage 4 CKD patient)
	**Technological factors**	
	Complexity	Q14: “… I think the [ePHR] system information should provide a complete explanation in simple and understandable language for our patients … but if the ePHR provides incomplete or vague explanations, the patient will be more worried than before.” *(A nephrologist)*
	Perceived usefulness	Q15: “In my opinion, for each patient, we should have a specific training page [in the ePHR] related to both the CKD disease [itself] and the underlying diseases … also, it [ePHR] should be based on the level of literacy of each person, it should display an educational video, audio, or textual information.” *(A nephrologist)*
	Perception of external control	Q16: “I think, only the patient should have access to the system … because it [ePHR] is a personal thing and the patient may not want to share his/her disease information with others.” *(A nurse)* Q17: “Because the patient's rights must be protected, the doctor and the patient must determine the level of access to the system for others…” *(A nephrologist)*
	Cost	Q18: “With such a system, I may not need to make an appointment with my doctor to get advice and ask questions … this will create a problem for the doctor's earnings.” *(A stage 4 CKD patient)*
	Relative advantage	Q19: “In all cases, the cost of counseling through this [ePHR] is less than the cost of face-to-face counseling. I come here from another town and one day of my life and also money is spent …” *(A stage 1 CKD patient)*
	Alternative strategies	Q20: “At this moment, if patients had any questions in dialysis wards, our nurses can answer them by telephone consultation. I think the [ePHR] system will work the same way, but with the difference that the patient can ask their questions through the system.” *(A nephrologists)*
	Compatibility	Q21: “I think access to this system will make our lives better, for example, our 10-year dialysis life will be 15 years … I think I will use it a lot in my daily life. For example, if this system tells us what diet we should have, it will be 100% useful.”*(A stage 5 hemodialysis CKD patient)*
	**Chronic disease factors**	
	Self-management	Q22: “I expect the [ePHR] system provide educational information about the [CKD] disease.” *(A stage 1 CKD patient)*
	Number of healthcare settings, healthcare providers and chronic illnesses	Q23: “…Patients in the first stages [of CKD disease] need different nutritional education information than dialysis patients … The medications they take vary in different stages … Catheter type, fistula, and special care are important for dialysis patients, but early stage patients do not need this information. In the early stages [of the CKD disease], laboratory tests may be repeated every month, but in patients with end-stage [of CKD] or other underlying diseases, laboratory tests may be different and should be recorded at shorter intervals … I think that applying these features [in an ePHR system] reduces the training load of [CKD] patients for the care team staff.” *(A nurse)* Q24: “I have kidney problem because of my lupus disease. In my opinion, it [ePHR] should contain information about all of our related problems too … Some patients have multiple diseases and like me they may have another doctor to visit …” *(A stage 1 CKD patient)*
	Access to care	Q25: “All patients do their dialysis at home after completing their training in this [peritoneal dialysis] ward. But some of them may have problems that they are not aware of or may forget our training material. So, they have to come to this hospital from their cities because we don't have another peritoneal dialysis center in our province … I think if their training is repeated through the [ePHR] system, it may reduce some of their problems.” *(A nurse)*
	Preferences for self-regulations	Q26: “… the [ePHR] system reminds me "you should go to the doctor today”, or says "tomorrow or two hours later is your appointment time”, or reminds me to take my test results, this is a good thing.” *(A stage 2 CKD patient)*
	Attitudes on negotiated collaboration	Q27: “I do not always have access to a doctor. I can use it [an ePHR] to send my medical records to a nephrology specialist for guidance and she can answer to my questions…” *(A stage 3 CKD patient)*
	Perceived complexity of condition and treatment	Q28: “… if a patient has a problem in an emergency and this feature is in the system [messaging feature], first of all, it will lead to higher patient expectation, and we cannot respond to them all the time. Secondly, it also increases the responsibility of the system; the patients may not be able to correctly tell us their problems [through ePHR], and we may give them incorrect recommendations, or patients may not correctly understand our recommendations [given through the ePHR], and [this] may cause problems for them.” *(Focus group meeting – A senior nephrologist)*
**UTAUT**	**Performance expectancy**	
	Perceived usefulness	Q29: “… Some of our [CKD] patients are not usually alert about their problems, also about their medications. They can’t give us much information about their ongoing treatments. These patients usually get hospitalized several times and then go under the diagnostic procedures again; [for example] the patient doesn’t know that has CT [CT-Scan results] … the patient doesn’t know what medications he is currently getting, and the patient is unaware of his/her drug allergies. If all of these are recorded in such a system [ePHR], we can easily reduce the amount of patient workload, reduce their lengths of hospital stay, reduce their costs, and maybe make our healthcare system more relaxed than what it is now.” *(A nurse)*
	Relative advantage	Q30: “When patients are discharged, their paper-based medical record is archived and the patient may not be hospitalized for another year. Retrieving these records from the medical records department is very time consuming and it takes time to figure out what has been done for the patient. But if this information is always made available for patients electronically [through the ePHR], we can access their medical history more quickly than usual.” *(A nurse)*
	Outcome expectations	Q31: “Well, I visit a lot of patients every day. So, when a patient comes back for a visit, I do not remember their previous treatments … I think with this [ePHR] system, I do know what has been our medication therapy approach, and what have been our patient's lab result trends.” *(A nephrologist)*
	Job-fit	Q32: “ I think this system provides us a time-saving tool, because patients can get answers to many of their questions through the educational section [ePHR]… we can then focus further on their care based on the information stored in their ePHR.” *(A nurse)*
	Extrinsic motivation	Q33: “Using this system makes our work faster. It will also be easier for us to track and compare patients' laboratory results.” *(A nephrologist)*
	**Effort expectancy**	
	Perceived ease of use	Q34: “… Some of the main patient information can be entered into the system from the CKD registration system … Also, the laboratory information of our patients is available in the HIS system. I think if such information is received from these systems, it will take less time for the information to enter the [ePHR] system.” *(Focus group meeting – A nurse)* Q35: “Answers can be prepared for some patients' questions. For example, what is my potassium level now and what should I do? The answer should be in the [ePHR] system … Some questions can be answered immediately without the need for an on-call nurse or physician.” *(A nephrologist)*
	Complexity	Q36: “If it is like the HIS system, we will enter all the information and everyone can use it easily.” *(A nurse)*
	Ease of use	Q37: “It sounds interesting, we can easily guide the patients, there is no problem at all. It will be very easy for me to work with these [ePHR] systems.” *(A nurse)*
	**Facilitating conditions**	
	Perceived behavioral control	Q38: “The advantage of this system is that there is no need to constantly review and search patient's paper records. Also, with this [ePHR] system, it will be easier to record and access patients' information. (A nurse) Q39: “…Other medical staff can view the information, but only patients, nephrologists and nephrology ward nurses can enter the information into the [ePHR] system.” *(Focus group meeting – A senior nephrologist)*
	Facilitating conditions	Q40: “… I think the level of access to the system can be determined by the doctor and the patient …” *(A nephrologist)* Q41: “…The patient may give us incorrect information. In my opinion, ensuring that the information in the [ePHR] system is properly understood by the patient is an important factor in its acceptance. Otherwise, the risk of transmitting incorrect information [of ePHRs] increases.” *(A nephrologist)*
	Compatibility	Q42: “Well, when we connect the patient to the hemodialysis machine, we do not have immediate and accurate access to the patient's serum phosphorus and potassium levels to determine the duration of hemodialysis … by having access to the [ePHR] system, we can do our job more accurately. Also, we don't need to spend time reviewing paper-based records …” *(A nurse)*
	**Social influence**	
	Subjective norm and social factors	Q43: “Everything should be planned beforehand… Who will be responsible to answer patients' questions? It is possible that a patient asks a question out of my specialty. Nurses can't answer every kinds of question. There should be a resident medical doctor to be responsive to these questions… there are questions about diet or medication side effects, nurses can respond to these kinds of questions, but what about whether or not the drug should be discontinued or [what to do with] the other problems with their drugs, physicians should respond to these kinds of questions…”. (an experienced hemodialysis nurse)

*PHRAM* Personal Health Record Adoption Model, *UTAUT* Unified Theory of Acceptance and Use of Technology, *ePHR* electronic Personal Health Record, *CKD* chronic kidney disease, *HIS* hospital information system, *CT-Scan* computed tomography-scan

### Factors that influence ePHR adoption by CKD patients

#### Personal factors

Our participants had positive attitudes towards the availability of an ePHR. They thought that it can increase their awareness of their disease and the care they needed (Q1). By removing temporal and geographical barriers, it can also facilitate access to care providers (Q2) and educational materials and health information (Q3), helping to alleviate patient stress (Q4) and expenses (Q5). Using such systems, patients expected to have access to a complete report of their condition anytime/anywhere (Q6).

Yet, many concerned that the advancing age (Q7), lack of interest (Q8), and low e-health literacy (Q9) would represent the main barriers to adopt the system. Similar barriers were also echoed during provider interviews. They mentioned that most of their patients have lower e-health literacy and therefore may not be able to understand or use these types of systems (Q10).

#### Environmental factors

Several patients reported their inability to use or access the technical equipment to use the ePHR (e.g., the Internet or smartphones) (Q9, Q11). They, however, believed that having supporters such as caregivers and their family members would assist them to bypass such barriers (Q11).

#### Technological factors

There was an agreement that the information in the ePHR should be displayed according to an individual patient's condition in a simple and easy to understand language and format (Q9). Thus, the use of complex medical terms and jargon should be avoided, because patients might misunderstand their disease condition or misinterpret information available within an ePHR (Q14). Participants believed that the availability of multimedia educational materials such as movies or even voice messages would facilitate ePHR’s acceptance and use, especially by patients with low e-health literacy levels (Q15). The next point was the importance of preserving patient privacy and information confidentiality and preventing unauthorized access to the ePHR (Q16). They recommended establishing mechanisms to strictly define the levels of access to protect privacy and information confidentiality (Q17).

Using such technology entails costs. Besides the cost of acquiring the technical equipment, patients were concerned that care providers might not support using the ePHR because it wasn’t clear how they would receive a fee for the service given through it (Q18). Therefore, some patients were willing to pay for this service rather than travel long distances and wait a long time to consult specialists (Q19).

#### Chronic disease factors

One of the main supporting functions for self-management, which all patients emphasized, was the need for education and training through an ePHR on the subjects inflicting chronic patients (Q22). Specialized, individualized, and purposeful information and educational materials (for different CKD stages and varying comorbidities) would increase patient awareness and reduce provider time and efforts spent on the education of subjects such as lifestyle, medication use, diet, and care in hemodialysis or peritoneal dialysis (Q23, Q24, and Q25). However, their care providers were cautious that simply making the information available within the system without any reasonable explanation might sometimes increase patient anxiety about their disease. They also requested a reminder system due to the chronicity of their disease and frequency of events and preventive measures, for example, to remind the time of medication use, upcoming follow-up visits, vaccinations, laboratory tests checkups, and notification of result availability (Q26). Table 3 provides examples of data categories requested by patients.

**Table 3:** The data categories (and items) required for support of CKD patients by an ePHR

Category			Data items
**Demographic information**	Patient demographics		First and last names, gender, national identification number, date of birth, marital status, job, contact numbers, address and postal code
	Demographics of patients’ care-givers		First and last names, gender, relation to the patient, emergency phone number, address, postal code
	Demographics of patients’ care providers		First and last names, types of care provider (e.g., nephrologist, nurse, internal medicine specialist, general practitioner/family physician, clinic secretary, medical intern or resident), work address, contact number
**History of patient's CKD**	Clinical history specific for CKD		The main cause of CKD, stage of the disease, the date of first CKD diagnosis, history of kidney biopsy
	History of RRT	General RRT information	Kidney transplant/dialysis status, the method of dialysis
		Clinical history of hemodialysis patients	Type of vascular access (including graft, fistula, temporary catheter or permacath), vascular access insertion date, start date of hemodialysis, frequency of hemodialysis per week, the address of hemodialysis unit
		Clinical history of peritoneal dialysis patients	Peritoneal catheter insertion date, start date of peritoneal dialysis, peritoneal dialysis frequency, exit-site status/infection, history of peritonitis
		Clinical history of diabetic patients	History of diabetes, diabetic retinopathy, diabetic ulcer (condition and place)
	General medical history	Lifestyle	Height, weight, history of alcohol use, history of smoking, other comorbid diseases
		Allergies	Medication allergies, food allergies, other allergies
	Hospital admission/medical procedure history	Internal units admission	Admission date, discharge summary
		Medical procedures	Result of the last echocardiography, admission date, proceadure summary
	Family history		First and last names, relation to patient, type of disease
**Diagnostic tests**	Biochemical tests		CBC, Creatinine, BUN, FBS, HbA1c, ALT, AST, ALP, Bili-T, Bili-D, TG, Chol, LDL, HDL, Calcium, Phosphate, iPTH, Vit D, Sodium, Potassium, Ferritin, TIBC, Iron, HBsAg, HCV Ab, TB, PSA, ESR
	Diagnostic radiology tests		KUB, CXR, CT-Scan, MRI, Sonography
**Medications**			Medication name, form, and dosage, time of medication intake, start time of medication, stop time of medication
**Training/education**			System user instruction, education materials for important medications, nutrition, lifestyle, and hemodialysis and peritoneal dialysis; training session for recommendation of family member screenings in specific conditions, frequently asked questions
**Daily reports**	Information of fluid intake limitation		Fluid intake volume and date, volume of urine, other fluid input/output volumes
	Daily clinical symptoms	Hypertension information	Systolic and diastolic blood pressure values with dates
		Glucose information for diabetic patients	Glucose values with time/date
**Scheduled information**			Date of an upcoming follow-up visit, date of due medical laboratory, date of due laboratory results, date of due vaccination

*CKD* chronic kidney disease, *RRT* renal replacement therapy, *CBC* complete blood count, *BUN* blood urea nitrogen, *FBS* fasting blood sugar, *HbA1c* Hemoglobin A1c, *ALT* alanine aminotransferase, *AST* aspartate aminotransferase, *ALP* alkaline phosphatase, *Bili-T* bilirubin total, *Bili-D* bilirubin direct, *TG* thyroglobulin, *LDL* low density lipoprotein, *HDL* high density lipoprotein, *iPTH* intact parathyroid hormone, *Vat D* vitamin D, *TIBC* total iron binding capacity, *HBsAg* Hepatitis B surface antigen, *HCV Ab* Hepatitis C virus antibody, *TB* tuberculosis, *PSA* prostate specific antigen, *ESR* erythrocyte sedimentation rate, *KUB* kidney, ureter, and bladder, *CXR* chest X-Ray, *CT-Scan* computed tomography-scan, *MRI* magnetic resonance imaging

While patients see an ePHR as an opportunity for greater communication with their care providers (Q27), care providers highlighted that synchronous communication through such a system should be minimal. Therefore, they recommended that patients should be educated to use the system only for their chronic conditions without the expectation of a prompt response from care providers, especially for acute conditions and emergency situations (Q28).

### Factors that influence ePHR adoption by CKD care providers

#### Performance expectancy

Our clinicians envisioned that easy access to patient latest medical information such as radiology and laboratory results (and their trends over time) and the ability to share them with other providers would save them time and prevent requesting extra tests (Q29 and Q31). They also valued accessing such data all the time. This would save them going back and forth to find necessary information across voluminous, but generally incomplete and illegible, paper-based records (Q30). To assess the reliability of data, they recommended displaying the source or an individual who entered different pieces of information. Clinicians believed that available education on the ePHR would reduce the amount of time they spend on patient education and training during regular visits, allowing them to spend more time on direct patient care (Q32).

#### Effort expectancy

A majority of clinicians envisioned that working with ePHR would be an easy task because of their prior experience with the HIS (Q34 and 36). They believed that the integration of the system to the HIS or the CKD disease registry would enable retrieving some basic patient information and test results, which would, in turn, reduce the amount of data entry time while increasing the accuracy of the information and facilitating ePHR adoption by different user groups. They also suggested considering a section for frequent questions and answers through the system to reduce the load of bidirectional communication (Q35).

#### Facilitating conditions

Our clinicians suggested considering two main user groups of patients and their family members, and care providers. They recommended full access to enter, view, and revise information for nephrologists and nephrology nurses but only permission to view information for other clinicians (e.g., internal medicine specialists, family physicians, residents, and interns in academic medical centers) (Q38 and Q39). Meanwhile, to protect patient privacy and information confidentiality, an authentication mechanism was overemphasized through predefined levels of access according to each user’s role in CKD care (Q40). Some of our care providers have concerned that the accuracy and reliability of the information provided by patients and/or other care providers through an ePHR should be given much attention, because, for instance, if patients enter incorrect information into an ePHR, it can lead to serious patient health risks (Q41).

## Discussion

In this study, we identified the requirements for an ePHR for CKD patients from users’ perspectives, and its content and structure needed to help support those requirements. These features were analyzed in the face of ePHR’s likely facilitators and/or barriers to its implementation and adoption. Experience shows that if such knowledge is applied for ePHR design, it can most probably contribute to a smooth ePHR implementation and adoption [26]. Overall, our participants expressed positive attitudes towards ePHRs and envisaged that a given ePHR will increase access to credible and trustworthy health information and individualized condition- and risk-based education. As recommended by a recent review, such an integrated patient-centered self-management approach with health literacy and information technology intervention will empower patients to self-manage their disease more effectively and can address the difficulties that are contributing to unsuccessful treatment outcomes in CKD patients [27]. Our participants emphasized that such a technology should be designed to fit the e-health literacy level of patients and only display CKD specific information depending on a patient’s individual conditions. To put it in other words, it should tailor information in ways that protect patients from being overwhelmed with extra, unnecessary, and stress-producing information (e.g. displaying care for infectious complications of the hemodialysis access or the pros and cons of kidney transplantation to an early stage CKD patient).

Studies have reported that one of the many benefits of ePHRs is easier access to patients' past and present medical data and information breaking through the time and space barrier and the ability to exchange and share them with whoever is involved in patient care [28–32]. According to our participants, ePHR can be a powerful tool to resolve problems with paper-based medical records such as their incompleteness, illegibility, and inaccessibility. It can also bypass time and space barriers to access care providers, for example, in cases when they do not remember the details of care recommendations or are puzzled about their roles and responsibilities in care plans. Such expectations from an ePHR are in line with the results of previous studies in which researchers reported improved patient-provider communication or where patients are reminded about what they will do for upcoming check-ups or vaccinations [33, 34]. Besides these, our participants also thought that the ePHR would save them time on patient education and information access that they would be able to spend that saved time on direct patient care, as reported previously [35]. Moreover, information access can potentially reduce unnecessary patient referrals for diagnostic or therapeutic procedures and lead to more timely decision making [36]. However, the findings on time saved via ePHR should be interpreted cautiously and the expectations kept modest because its use might also be perceived as a time-consuming task. This is because some studies reported that using ePHRs by providers required their duplicate time and efforts to handle issues related to the ePHR use parallel to the other tasks of patient visits in the office time [35, 37]. To counter for such after effects and pave the path to success, developers should carefully consider the socio-technical and organizational aspects of healthcare e.g., the characteristics of users and the integration of ePHRs into their workflows as well as their integration into the available health information technology (HIT) infrastructure such as Electronic Medical Records (EMR) and Electronic Medical Records (EHR) [6, 38].

Management of CKD is based on stopping the renal damage or at least slowing its progression through monitoring kidney function, modifying risk factors (e.g., hypertension and diabetes), and preventing patient cardiovascular complications [39, 40]. Like other chronic conditions, effective CKD management requires patient partnership with care providers through self-management practices in order to set goals, solve problems, manage symptoms, decide on optimal and personalized action plan, and implement that plan effectively [15, 41, 42]. A meta-analysis has shown that self-management interventions in CKD care is beneficial for decreasing urine protein excretion, controlling blood pressure, and life style modifications (such as exercise capacity) even in the early stage CKD patients [15]. ePHRs are increasingly seen as transformational agents in developing and nurturing productive interactions between patients and their healthcare providers towards a patient-centered model of care [43, 44]. Our participants emphasized on the notion of patient-centered model of care and discussed that an ePHR would be embraced and valued, if it is developed based on CKD patients’ needs and requirements such as providing personalized education and counseling on the basis of a patient’s health condition and risk factors, health literacy and disease progression levels. It is noteworthy to mention that CKD patients are spanned across all ages, disease stages, and literacy levels. To put in other words, there are both younger CKD patients in ESRD and elderly patients in earlier stages. Therefore, a mindfully developed ePHR should be used as a teaching tool and learning strategy to accommodate the differing self-management needs and requirements of these entire patient groups based on their age, literacy levels, and disease stages [45–47]. To this end, developers should improve the performance of ePHRs for long-term use by engaging patients and offering added values for them to self-manage CKD disease trend. This would be better achieved by involving potential user groups in all phases of system development to ensure ePHRs match with the capacity of existing infrastructures and the literacy and cognitive abilities of their intended users.

Several reviews have so far studied the barriers to ePHR adoption [6, 48–51]. These studies have reported providers’ concerns about legal issues to respond to patient inquiries through ePHRs (e.g., who from the members of a care team seeing a patient’ inquiry would respond to an emergent situation) and also concerns over the privacy and security of information. Other barriers were also discussed in the literature such as patients’ individual factors (e.g., patient age, gender, health and computer literacy, and access to computers and the Internet), technical factors (e.g., lack of interoperability with other Health Information Technology (HIT) systems such as electronic health records, and lack of customized features for chronic conditions) [50–52]. Many of these issues were also raised by our participants as concerns that needed to be considered before any ePHR adoption. For example, they discussed the feasibility of automatically retrieving information stored in a CKD registry or HIS in order to reduce workload associated with data entry, as previously reported as a critical point especially in untethered ePHRs [35, 37, 53]. Therefore, easy, intuitive, and preferably automatic data entry for some data should be taken into account when designing such systems. Our participants also emphasized the need to preserve the privacy and confidentiality of data. Similar concerns have also been reported in other studies, for example, patients had been concerned about the confidentiality of a stigmatized or sensitive condition such as having depression or infection with Human Immunodeficiency Virus, or others were worried that their information could be accessed and might be misused by insurance companies to deny coverage [54–56]. This was also the case for *caregivers*’ access to some sensitive information [55, 57, 58]. Participants also highlighted the cost of services delivered through ePHR. Besides the necessity for tangible incentives in order to adopt PHR, it has been shown that clarity is needed for how PHR-related services would be paid for, who would pay (patients or payers), and under what circumstances [53, 59–61]. Such findings show that the successful application and use of ePHRs should address likely barriers before any implementation, or otherwise, these may threaten its deployment.

Parallel to barriers, we also considered likely facilitators, which might contribute to the ePHR’s smooth implementation; and compared our findings with the others [48, 49, 62–64]. A review found that patient encouragement by providers to use ePHR, their perception of control over health information, and also their perception of ePHR’s greater potential to improve patient-provider communication are facilitators that can help expedite ePHR adoption [49]. Similarly, our participants valued ePHR features such as facilitated communication with providers and easier access to data. Designing a system to satisfy these expectations would likely promote its adoption. Another review reported that besides the availability of secure messaging and eVisits with care providers, online appointment scheduling and reminders, and online access to patient laboratory and radiology results also promote patients to use ePHRs [64]. Participants also requested the availability of care reminders (e.g., about the time to take medications or to receive a vaccination shot) and online availability of laboratory results and their trends because they believed that these features would promote self-management. Our findings also accord with those of others in which tailored system interfaces to the educational and e-health literacy level of patients are recommended for ease of use [62]. Moreover, it also became evident in our study that caregivers should be considered as users and involved in the PHR application as surrogates to patients in order to overcome some of the predicted barriers such as health and computer literacy by elderly, as recommended in the literature [63].

In the deployment of HIT systems, their data elements and design structure can influence what advantages they would be able to deliver. In a CKD study in Canada, data elements were classified into four main categories of demographics, educational, behavioral and activity monitoring, and laboratory results [18]. Researchers in Thailand reported a knowledge-based ePHR system, especially designed for CKD patients, which had electronic forms for recording personal information, medications, laboratory tests, dietary patterns, and exercise activity [17]. In our study, besides the above data categories, participants requested the following categories to be included in the system as well: a comprehensive, purposefully documented medical history, patient’ scheduled reminders, individualized educational contents, and information on record access by caregivers and care providers. When compared with the results of two previous CKD studies, data items associated with the recording of behavioral and activity monitoring, diet, and exercise were not included in our final list, mainly because they were not approved for the early implementation phase. Participants believed that the system to start with should be much simpler with the most important items individualized based on each patient’s health conditions in order to be manageable. Extra functions can be added step-by-step later on when patients get acquainted with the system. It was also decided that the message exchange function between patients and care providers should be only for non-emergency situations; and in emergencies, patients should be trained to contact care centers to get the required care, promptly. This was because of concerns that messaging through the system may induce the perception that somebody is available all the time to respond even to an emergency condition. This finding and other chronic disease factors have rarely given full attention in the literature [65]. Our findings that the intended ePHR should include functions and structures such as messaging, reminders, disease-specific individualized educational information, and medical record summary, have also been similarly reported elsewhere [17–19]. Meanwhile, ePHR is an evolving technology and it should be borne in mind that access to personalized, disease-specific information through a robust architecture, controlling secure access to patient information, should be at the center stage of the design efforts [66, 67].

### Strengths and limitations

In the present study, we used nephrology care as exemplary chronic disease care with all its corresponding characteristics such as CKD patient’s multiple comorbidities, underlying diseases, and disease stages with various information/educational needs along the way, multiple patient hospital admissions/patient referrals, and the involvement of different kinds of care providers in different types of wards/units/settings, to get a deeper insight into what requirements/need an ePHR should cover in order to provide value for both chronic disease patients and their care providers involved in this complex chronic disease care. Therefore, the approach used in this study is fairly applicable for the analysis of requirements in other chronic diseases. However, it has limitations that deserve to mention here. Although our results provide a thorough overview of CKD care involving different stakeholders with an ePHR, its specific care requirements may not be generalizable to other nephrology settings. For example, our nephrology center was the pioneer in the design and implementation of a CKD registry in the country based on its care context [68]. Several universities have just started negotiations for this registry’s implementation. Hence, it is possible that the emphasis to integrate an ePHR with the available HIT systems would not be immediately feasible in other nephrology settings. Next, this study was conducted in the adult nephrology care; therefore, its findings may not be applicable in the pediatric nephrology care in which a patient’ parents commonly deal with ePHRs.

## Conclusions

This study is one of a few CKD-ePHR studies that specifically focused on the system requirements of chronic patients and their care providers with a bird’s eye view of CKD care. According to our participants, an ePHR was perceived as a useful tool in improving the self-management of CKD patients by providing better patient information and improved patient-provider communication. If in use, patient lower health and technology literacy and the high workload of care providers will represent some of the main barriers to ePHR adoption, which should be mindfully considered beforehand. Drawing upon the facilitators identified in this study, and of the others' as well, may help to overcome some of the barriers and increase users’ acceptance.

To conclude, according to the findings in the current study, we need to develop a patient-centered ePHR that is tailored to the needs of all its users in daily, short- and long-term use, especially in the context of chronic care. Meantime, chronic care has unique requirements that should be considered. Given the multiplicity of users having varying roles, and locating in different units, it would be helpful and more productive if all are involved in the early stages of system development and implementation in order to inform the design regarding each one’s requirements. We hope that the insights gained from this study can be helpful for designers, implementers, and researchers of such systems in supporting patients in self-management in an orchestrated effort with their care providers.

### Contributions to the literature

- Fitting ePHRs within the daily practices of care providers and patients, especially in chronic kidney disease care requires user-centric design.
- To do so, understanding users’ needs and requirements regarding the content, and functions of an ePHR is a prerequisite.
- Fully understanding users’ needs and requirements can help to design an ePHR system that better supports users in their socio-technical context of chronic kidney disease care and enable empowering chronic kidney patients in self-management.

## Supplementary Information

**Additional file 1**: Appendix 1. Interview and focus group questions.

## Data Availability

The datasets used and/or analyzed during the current study are available from the corresponding author on a reasonable request.

## Abbreviations

ePHR: Electronic personal health records
CKD: Chronic kidney disease
EHR: Electronic health records
EMR: Electronic medical records
UK: United Kingdom
US: United States
HIS: Hospital information system
PHRAM: Personal health record adoption model
UTAUT: Unified theory of acceptance and use of technology
CT: Computed tomography

## Footnotes

1 The number of Qs is according to the order of their appearance in Table 2.
