# Supplementary material for: Patient empowerment through a user-centered design of an electronic personal health record: a qualitative study of user requirements in chronic kidney disease
Source: BMC Med Inform Decis Mak. 2021 Nov 24;21:329. doi: 10.1186/s12911-021-01689-2 (PMC8611831; doi:10.1186/s12911-021-01689-2)
Supplement: Supplementary file 1 — Additional file 1: Appendix 1. Interview and focus group questions. [file 12911_2021_1689_MOESM1_ESM.docx]

**Appendix 1:**

| **Interview questions** | |
| --- | --- |
| **CKD patients' interview questions** | - What information do you have about the electronic personal health record (ePHR)? - In your opinion, how can this system help you manage your CKD patietns and improve the care you provide? - In your opinion, what information will help you better manage your disease using this system? - In your opinion, what system-based reminders might be useful to better manage your disease? - In your opinion, what are the advantages of managing the disease through using this system compared to your direct hospital visits? - In your opinion, what are the disadvantages of managing the disease through using this system compared to your direct hospital visits? - Would you prefer to check your condition through an ePHR or have it explained to you at the hospital? - What information do you think you need to share with your doctor during your visits? - In your opinion, what other help (besides your previous talk) can be given to using ePHR? - In your opinion, what are the barriers to use such systems? - What solutions can you offer to overcome these barriers? |
| **Care providers' interview questions** | - Do you have any information about ePHR? - In your opinion, how can using an ePHR help to provide care (nursing/medical) to CKD patients? - In your opinion, what can the benefit of using an ePHR be compared to patients’ paper records? - What problems do you think there might be for the patients if they use an ePHR?   - How can we manage these problems?   - How much do you want to communicate with your patients through an ePHR? - What kinds of information would you prefer to see in this system? - How do you want to display this information in your user interface? - In your opinion, what diet information should this system contain? - Who can (among patients' care providers) be the primary users of the ePHR and who can answer patient questions? - Who can view patient information? - What are the general categories of information that should be included in an ePHR to help you understand your patient's condition? - A CKD patient may also have other comorbidities besides CKD, which type of disease information do you need for follow-up? - In your opinion, what kinds of information do CKD patients need in the early stages of the disease? - What information do you think dialysis patients need? - What information do dialysis patients need to enter to provide them with better care through an ePHR? - In your opinion, what information is needed for patients undergoing dialysis at home? |

| **Focus group questions** |
| --- |
| - According to the information extracted from the interviews, what kinds of information/contents would you prefer that both patients and care providers see/access in this system? - According to the information extracted from the interviews, what kinds of information/contents don’t you like that both patients and care providers to see/access in this system? Why? Please explain… - According to the proposed structure of ePHR extracted from the interviews, which functions you prefer to be designed/implemented in the early stages of system development? - According to the proposed structure of ePHR extracted from the interviews, which functions don't you like to be designed in the early stages of system development? Why? Please explain… - According to the barriers identified from the interviews, which facilitators or solutions can you recommend to overcome such barriers, while develop, implement or use of the proposed ePHR? Please explain… |
